# Discharge‐Targeted Hydraulic Tomography to Quantify and Locate Aquifer Discharge

**DOI:** 10.1111/gwat.70081

**Published:** 2026-05-15

**Authors:** Konstantin Drach, Carsten Leven, Olaf A. Cirpka

**Affiliations:** ^1^ Department of Geosciences University of Tübingen Tübingen Germany; ^2^ Cluster of Excellence (EXC 3121): TERRA – Terrestrial Geo‐Biosphere Interactions in a Changing World University of Tübingen Tübingen Germany

## Abstract

Quantifying and localizing groundwater discharge is inherently difficult. It requires knowledge about hydraulic conductivity and the hydraulic gradient on the scale of interest. Conventional hydraulic testing, such as pumping tests, may fail in the presence of heterogeneity and complex structural boundaries. While advanced 2D and 3D hydraulic tomography may resolve small‐scale heterogeneity, it is typically limited to small spatial scales and requires costly field installations. We propose a simplified tomographic approach using a limited number of pumping and observation wells spatially distributed over a well profile in the order of 100 m transverse to the direction of ambient flow. To infer the spatially variable hydraulic‐conductivity field from drawdown data with its uncertainty, we apply an iterative ensemble smoother. Subsequently, the posterior ensemble of hydraulic‐conductivity fields is used to calculate total and specific discharge based on the observed ambient hydraulic heads in the same wells. We test our approach in a synthetic scenario mimicking a channel‐like aquifer such as the quaternary fill in a small river valley. The results demonstrate that multiple spatially distributed pumping tests are suitable to quantify total discharge and its associated uncertainty. The approach is more reliable than a conventional one that estimates effective transmissivity from fitting analytical solutions to pumping‐test data. The tomographic analysis additionally allows locating spatial patterns of specific discharge at a resolution similar to the spacing of the wells, which may be important when assessing and remediating contaminant plumes.

## Introduction

Hydraulic conductivity links the gradient of hydraulic head to specific discharge. On scales relevant for water management, however, estimating groundwater discharge is hampered by aquifer heterogeneity. While hydraulic heads are straightforward to measure, determining a heterogeneous conductivity field is less trivial. For the total discharge passing through the section of an aquifer with uniform‐in‐the‐mean hydraulic gradient a flow‐effective conductivity value can be defined, which determines the proportionality between the large‐scale flux and the mean hydraulic gradient. The flow‐effective conductivity is bounded by the harmonic and arithmetic means of the conductivity field (Batchelor 1974). These limits are the exact values for perfectly stratified aquifers with the uniform hydraulic gradient being orthogonal and parallel to the stratification, respectively. The geometric mean is the effective value for an infinite second‐order stationary, multi‐Gaussian log‐conductivity field with isotropic correlation function, obtained at scales much larger than the integral scale of the heterogeneity (Matheron 1967). While these theoretical considerations are good for system understanding, they are of limited value in practical applications because the true spatially distributed conductivity field is typically so poorly known that geostatistical characterization is severely hampered, multi‐Gaussianity and stationarity may be questioned, and boundary conditions or preferential flow paths may lead to strong deviations from uniform‐in‐the‐mean hydraulic gradients (e.g., De Marsily et al. 2005; Zhou et al. 2014; Tahmasebi 2018; Moore et al. 2022).

The conventional approach of obtaining the hydraulic properties of an aquifer is to perform a hydraulic test, such as a pumping test, and analyze it by fitting an analytical solution of the groundwater‐flow equation for a homogeneous medium to the data. Strictly speaking, pumping tests using fully screened wells only determine transmissivity that is the vertical integral of hydraulic conductivity over the aquifer thickness. Nevertheless, we assume that the thickness is known and constant, and that vertical variations of conductivity do not affect the horizontal flow patterns. That is, we focus on horizontal heterogeneity for which pumping tests and analytical solutions have been proven to be a valuable tool. Sanchez‐Vila et al. (1999) found that the conductivity obtained by single‐well pumping tests in heterogeneous aquifers with a multi‐Gaussian log‐conductivity field indeed converge to the geometric mean when analyzing the late‐time drawdown, while the apparent storativity values show strong fluctuations. Leven and Dietrich (2006) demonstrated that a combination of single‐well pumping tests can be used to assess spatial anomalies of a conductivity field. They also showed that multi‐well setups are sensitive to larger and therefore more representative volumes despite being more ambiguous than single‐well pumping tests. Dann et al. (2008) combined the analytical analysis of multiple drawdown curves, measurements of active tracer breakthrough curves, and grain size analysis to assess the effect of permeable channels on the overall water and solute discharge. Using a two‐well setup, Larocque et al. (2009) reliably inferred the effective conductivity and corresponding flow rates in a virtual second‐order stationary aquifer with sufficiently long pumping durations. However, the analytical solutions used in the classical interpretation of pumping tests are based on simplifying and thus restricting assumptions. The solution of Theis (1935), typically used to interpret transient pumping tests with a single pumping and a single observation well, assumes an infinite, homogeneous, confined aquifer. No real aquifer truly meets these assumptions. While many extensions of the Theis (1935) solution exist (e.g., for a sloping aquifer bottom, straight boundaries, uniform anisotropy, leaky aquifers, two‐layered systems etc., Kruseman et al. 1990), none of them can address natural heterogeneity. Hence, hydraulic‐conductivity values derived by conventional analysis of pumping‐test data can only be seen as weighted spatial averages, where the weighting function crucially depends on the hydraulic conductivity and storativity distributions themselves and the time since the beginning of pumping (e.g., Sanchez‐Vila et al. 1999; Leven and Dietrich 2006; Manewell et al. 2023). The conceptual shortcoming is therefore that averaging volumes need to be sufficiently large to be representative for a targeted volume of interest (ergodicity assumption) while being unknown a priori. In other words, a heterogeneous aquifer needs to be homogeneous on the scale of the sensitivity volume of a pumping test.

Rather than determining a single effective hydraulic conductivity, hydraulic tomography aims at resolving its heterogeneity using a multi‐well setup with a suite of combinations of pumping and observation wells. Substituting the obtained hydraulic‐conductivity field into models of ambient flow, hydraulic tomography allows estimating the spatial distribution of specific discharge, which is of interest in applications such as the assessment of solute transport. A comprehensive summary of early progress in hydraulic tomography is given by Cardiff and Barrish (2011). Numerous field applications of the method have been conducted (e.g., Cardiff et al. 2012, 2013a; Hochstetler et al. 2016). The information content of hydraulic‐test data about spatial patterns of hydraulic conductivity is limited due to the diffusive nature of groundwater flow resulting in inherent ambiguities (e.g., Illman et al. 2008; Bohling and Butler Jr. 2010). To overcome these limitations, multiple approaches have been proposed and tested. Doro et al. (2015), Somogyvári and Bayer (2017), Sánchez‐León et al. (2020), among others, included data of solute or heat tracers. Another approach is to perform oscillatory extraction and injection of water to stress the aquifer (Cardiff et al., 2013b; Cardiff et al. 2020). Aliouache et al. (2025) demonstrated that oscillatory signals with short periods (1 min) lead to a better characterization of transmissivity and storativity fields compared to constant‐rate pumping tests. The authors also showed that oscillatory pumping tests are less sensitive to hydraulic‐conductivity anomalies outside of the investigation volume. Despite the inherent limitations of hydraulic tomography, multiple studies have recently demonstrated its applicability to fractured media, investigating hydraulic and geometric properties of connectivity features (e.g., Mohammadi and Illman 2019; Klepikova et al. 2020; Tiedeman and Barrash 2020; Ringel et al. 2021). Karstic features could be mapped by Duan et al. (2022) who were able to distinguish fractured and karstic hydrofacies from the surrounding matrix. Combining hydraulic tomography, tracer tests and hydraulic‐head observations under ambient‐flow conditions, Qian et al. (2024) could identify preferential flow paths in ambient flow along known tectonic faults in a coal‐mine setting. Mtsetfwa et al. (2025) illustrated that no‐flow boundary conditions manifest as low‐conductivity anomalies in tomography results when erroneous constant‐head boundary conditions were assumed. The current state of experience with hydraulic tomography is hence promising. With boundary conditions, conductivity structures and in particular connectivity features such as fractures, faults or karstic pathways, all controlling factors of discharge through an aquifer, can and have been targeted by hydraulic tomography.

The logistic effort of hydraulic tomography is quite high. As a consequence, most studies using this technique have been performed in the context of research projects and were restricted to small domains with length scale on the order of 10 m. In order to resolve all individual features of heterogeneity, at least one well per integral scale is necessary in each spatial dimension, which quickly becomes logistically unfeasible on larger scales. Furthermore, hydraulic tomography studies typically focus on the spatial distribution of hydraulic conductivity only. However, hydraulic conductivity is merely a material parameter relating boundary conditions to state variables of interest, such as hydraulic heads, flow velocities, and solute concentrations. While perfect knowledge of the hydraulic‐conductivity field throughout the aquifer in combination with perfect knowledge of hydraulic boundary conditions would lead to perfect knowledge of the velocity field, less information may be needed when addressing a more specific question.

The present study aims at quantifying the total discharge passing through an aquifer and identifying the variability of specific discharge in a profile perpendicular to the mean direction of flow. Such knowledge might be crucial for decision making when it comes to mitigating a contaminant plume or designing a hydraulic barrier for protection of a vulnerable site. While the definition of single flow‐effective conductivity values by classical analysis of pumping tests depends crucially on factors that are unknown a priori (i.e., heterogeneity type and sensitivity volumes), finely resolved conductivity fields derived by hydraulic tomography might neither be used to link hydraulic heads and total discharges if their extent is too small in a given hydrogeological context. This highlights the need for adapting hydraulic tomography toward a more target‐oriented approach: Rather than resolving the hydraulic heterogeneity per se, only those features need to be identified that influence the targeted property of the aquifer or the flow field on the desired scale. Deliberately sacrificing fine‐scale resolution by large spacing of pumping and observation wells might allow increasing the investigation volume without excessive effort. In other words, the purpose of the analysis is shifted from reproducing the true underlying subsurface features toward the ability to predict a designated target (total and specific discharge). This aligns with the previously proposed approach of prediction‐specific model design which aims to tailor models and their data assimilation only for designated metrics and/or hypothesis testing (Doherty and Moore 2020).

In the current study, we investigate how hydraulic tomography may be applied along a control plane of a width on the order of 100 m to infer specific and total discharge across this control plane from the obtained conductivity field and measured ambient hydraulic heads. We call this approach “Discharge‐Targeted Hydraulic Tomography.” Test data are generated synthetically from a generic 2D exemplary reference case. A respective field setting, in which the required wells can be installed with reasonable costs, may be a shallow channel‐like aquifer such as the Quaternary filling of a small river valley when direct‐push equipment is utilized for well installations. We apply a stochastic framework to quantify the associated uncertainty. The tomography results are subsequently compared to those resulting from traditional methods of analyzing drawdown curves based on the Theis (1935) solution.

## Method

### General Approach

The concept of hydraulic tomography is to stress an aquifer at different locations and observe the response of hydraulic heads at multiple locations. Typically, pumping tests are conducted, and drawdown curves are measured and inverted to obtain the spatial distribution of (log‐)hydraulic conductivity. In inversion, hydraulic‐conductivity values (and potentially other parameters such as specific storage) are adjusted to meet the measured drawdown data. This requires a predictive model that solves the groundwater‐flow equation to link an assumed conductivity field to a simulated data vector. Because hydraulic conductivity is a continuous spatial field, the number of discrete conductivity values is theoretically unlimited, whereas the number of observations is finite, leading to an ill‐posed optimization problem. This can be overcome by parameterizing the conductivity field, (e.g., by the introduction of distinct zones, Carrera and Neuman 1986), other trend functions with unknown trend coefficients, and/or by regularization. In regularization, the objective function representing the misfit of the data is amended by a term penalizing the variability of the estimated field. In most cases the regularization term has a quadratic form, such as the sum of squared differences to a prior mean value (dampening), the sum of squared discretized gradients (smoothing), or the negative log‐likelihood of a multi‐Gaussian prior distribution (Kitanidis and Vomvoris 1983; Kitanidis 1996; McLaughlin and Townley 1996; Benning and Burger 2018). The merger of a data‐misfit and a regularization term can be derived from Bayes' theorem, putting parameter estimation into the framework of conditional statistics (e.g., Evensen and Van Leeuwen 2000).

In order to investigate the discharge through an aquifer of approximately 100 m width, we propose to establish a control plane *A* [L^2^] perpendicular to the direction *x* [L] of the expected mean flow. Then the total discharge *Q* [L^3^T^−1^] through *A* is:

(1) $$Q=\int_{A}-K\left(x\right)\frac{\partial h\left(x\right)}{\partial x}\mathrm{dA}=\int_{A}q_{x}\left(x\right)\mathrm{dA},$$

where *K* [LT^−1^]is hydraulic conductivity, *h* [L] is the hydraulic head, and *q*_*x*_ [LT^−1^] is the specific discharge in the *x*‐direction. The target of most hydraulic‐tomography studies is *K*(**x**), whereas the targets of the current study are *Q* and *q*_*x*_(**x**) at the control plane, which requires *K*(**x**) within the control plane and ambient hydraulic‐head values across it.

**Figure 1:**
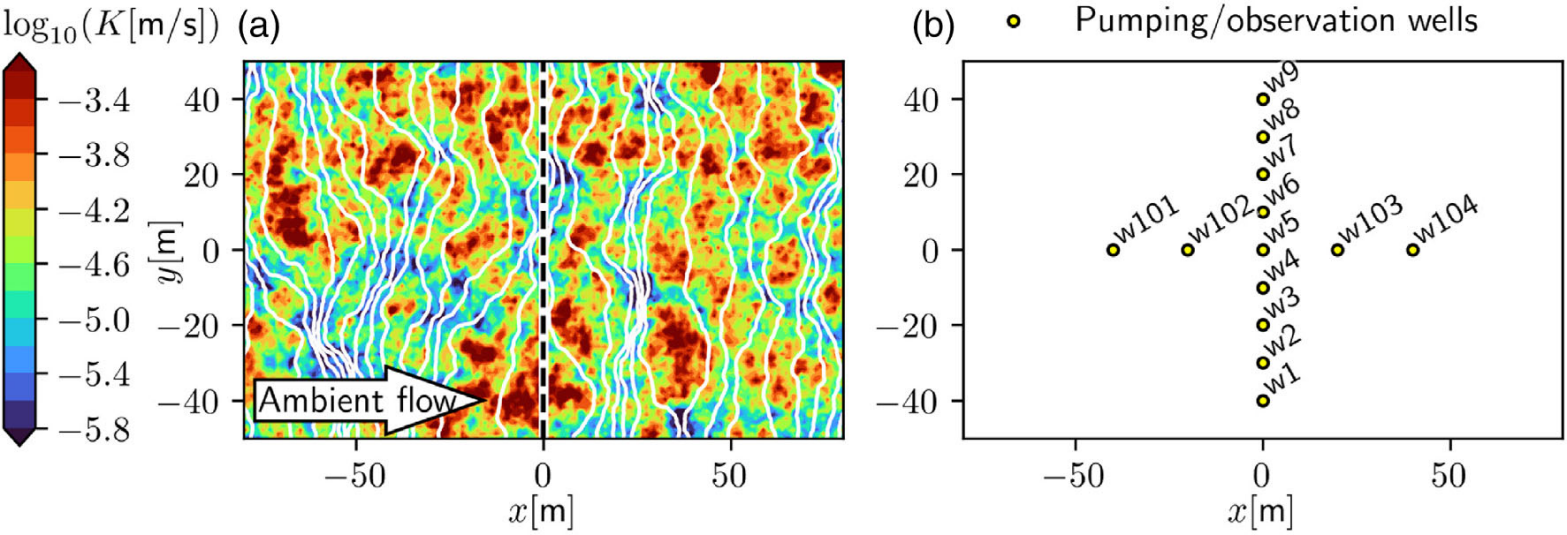
(a) Reference log‐hydraulic conductivity field and contour‐lines of hydraulic head (white lines) for ambient flow. No‐flow conditions are applied at the boundaries at the top and bottom. The central dashed line indicates the defined control plane. Buffer zones (15 km) are applied up‐ and downstream of the domain shown. (b) Location of wells.

In order to infer *K* along the control plane, we suggest a corresponding profile of wells with a spacing on the order of 10 m. A small number of longitudinally arranged wells extend the arrangement in the second horizontal direction. Such a design is illustrated and tested below (Figure 1). Installing observation wells along control planes has previously been done by other authors (e.g. Dann et al. 2008). However, to our knowledge this was never done in the context of hydraulic tomography. Our well placement is coarser than in most hydraulic‐tomography studies, which may limit the spatial resolution, but we want to keep the logistic efforts of a respective field experiment within reasonable limits while covering the entire width of the aquifer. For practicality, we use a 2D domain assuming horizontal flow to be only marginally affected vertical heterogeneity. Three objectives are targeted with this arrangement. First, by choosing large spacings between pumping and observation wells (10 m) only flow‐relevant features should be captured rather than small‐scale heterogeneity. In this context, flow‐relevance refers to those anomalies of hydraulic conductivity that are necessary to relate observations of hydraulic heads and total discharge on the chosen length scale (in this case 100 m). Second, the transverse width of the area of interest is entirely covered so that the measured data is sensitive to all such anomalies on the respective spatial scale. And third, measurements of ambient hydraulic heads in the wells need to capture the hydraulic gradient in the main flow direction and local gradient variations due to flow relevant anomalies. Such a setup might be further improved by optimal‐design methods (e.g., Nowak and Guthke 2016), which is, however, beyond the scope of this study.

### Governing Equations

Under confined conditions, both ambient groundwater flow and flow to an extraction well are described by the groundwater flow equation for confined conditions:

(2) $$S_{s}\frac{\partial h}{\partial t}-\nabla \left(K\nabla h\right)=G,$$

where the specific storage *S*_*s*_ [L^−1^] and the hydraulic conductivity *K* [LT^−1^] are spatially distributed aquifer properties. The hydraulic head *h* [L] is the main state variable and *t* [T] denotes time. *G* [T^−1^] is a source/sink term representing groundwater recharge or extraction/injection of water. The vector field of specific discharge **q** [LT^−1^] describes the volume of water flowing through a unit area perpendicular to it. It meets Darcy's law:

(3) q=−K∇h.

We consider that ambient flow is at quasi steady state, in which the time derivative of hydraulic head is negligible. In a transient pumping test, the drawdown *s* [L] is defined as the change in hydraulic head since the start of pumping at time zero:

(4) s(t)=h(0)−h(t)

The groundwater‐flow equation is subject to boundary conditions and to an initial condition in the transient case. In our simulations, we use the finite‐element code *HydroGeoSphere* (Brunner and Simmons 2012) to solve Equation 2.

### Second‐Order Stationary Random Log‐Conductivity Fields

We assume that the log‐hydraulic conductivity field, log_10_(*K*) is the outcome of a second‐order stationary random space process, characterized by variations across the entire domain that are spatially correlated according to a stationary covariance function $C\left(x_{1}-x_{2}\right)$ (Hoeksema and Kitanidis 1984):

(5) $$\begin{array}{ll} C\left(x_{1},x_{2}\right) & =E\left[\left(\log_{10}\left(K\left(x_{1}\right)\right)-\mu_{\text{logK}}\right)\left(\log_{10}\left(K\left(x_{2}\right)\right)\right.\right.\\ & \;-\left.\left.\mu_{\text{logK}}\right)\right]=\sigma_{\text{logK}}^{2}\rho \left(d\left(x_{1},x_{2}\right)\right), \end{array}$$

where *μ*_log*K*_ and *σ* ^2^_log*K*_ are the spatially uniform mean and variance of log_10_(*K*) and *ρ*(·) is the correlation function that only depends on a distance metric *d* [L] between locations **x**_1_ and **x**_2_ [L]. In this study we mostly use the Matérn covariance function (Rasmussen, 2004) of order one, which leads to differentiable realizations of the log‐conductivity field while showing long‐range correlations:

(6) $$\rho \left(d\right)=d\cdot K_{1}\left(d\right),$$

where *K*_1_(·) is the first‐order modified Bessel function of the second kind. For simplicity we assume an isotropic correlation function, where *d* is the Euclidean distance scaled by a characteristic length *l* [L]:

(7) $$d=\frac{{\left|\left|x_{1}-x_{2}\right|\right|}_{2}}{l}$$

Under these assumptions the spatial statistics of log_10_(*K*) are fully defined by the mean *μ*_logK_, variance *σ* ^2^_logK_, and characteristic length l.

### Inverse Kernel: The Iterative Ensemble Smoother (IES)

In order to infer the log‐conductivity values at computational cells, we apply the iterative ensemble smoother (IES) in its Levenberg–Marquardt formulation (Chen and Oliver 2013), which is a stochastic inversion algorithm based on Bayes' theorem assuming a multi‐Gaussian prior and a multi‐Gaussian likelihood. Unlike the standard Ensemble Kalman Filter (EnKF, Evensen 1994), it performs the parameter update using all data at once instead of successively assimilating the measured data as they become available. To account for a nonlinear dependence of the measurable quantities on the parameters, the IES performs successive linearization about the current estimate. In contrast to classical Gauss‐Newton approaches like the quasi‐linear geostatistical approach (Kitanidis 1995), the method does not require explicitly calculating the sensitivities of all measurements with respect to all parameters. Instead, all necessary matrices are evaluated from ensembles of parameters and associated model outcomes. A detailed rigorous derivation of the IES from Bayes' theorem and the standard Ensemble Kalman Smoother EnKS is given elsewhere (Van Leeuwen and Evensen 1996; Chen and Oliver 2012; Chen and Oliver 2013). Here, we restrict ourselves to a summary of the most relevant aspects.

Vectors of model parameters and measured data are considered to be random, correlated variables represented by an ensemble of $N_{e}$ realizations. Before considering the measurements in a vector $d^{0}$, the parameters follow a prior distribution that is approximated by a prior ensemble of vectors $m_{j}^{\mathrm{pr}}$ with $j=\left\{1\ldots N_{e}\right\}$. The data and prior‐parameter vectors are both assumed to follow a multi‐Gaussian distribution. Under these assumptions a vector of model parameters $m_{j}$ that minimizes the objective function

(8) $$\begin{array}{ll} S\left(m_{j}\right) & =\frac{1}{2}{\left(m_{j}-m_{j}^{\mathrm{pr}}\right)}^{T}C_{M}^{-1}\left(m_{j}-m_{j}^{\mathrm{pr}}\right)\\ & \;+\frac{1}{2}{\left(g\left(m_{j}\right)-d_{j}^{0}\right)}^{T}C_{D}^{-1}\left(g\left(m_{j}\right)-d_{j}^{0}\right) \end{array}$$

corresponds to the maximum posterior likelihood according to Bayes' theorem. Here, $C_{M}$ and $C_{D}$ are parameter and data covariance matrices, respectively, g(m) denotes the forward model operator and $d_{j}^{0}$ is the measurement vector $d^{0}$ randomly perturbed by the assumed measurement uncertainty.

The IES minimizes $S\left(m_{j}\right)$ by iteratively updating $m_{j}$ starting from $m_{j}^{\mathrm{pr}}$:

(9) $$\begin{array}{ll} \delta m_{j} & =-{\left[\left(1+\lambda \right)C_{M}^{-1}+G^{T}C_{D}^{-1}G\right]}^{-1}\\ & \;\times \left[C_{M}^{-1}\left(m_{j}-m_{j}^{\mathrm{pr}}\right)+G^{T}C_{D}^{-1}\left(g\left(m_{j}\right)-d_{j}^{0}\right)\right] \end{array}$$

where $\delta m_{j}$ is the difference of the vector $m_{j}$ from one iteration to the next and G denotes the Jacobian, that is, the sensitivity between data and parameters linearized about the current estimate for a given forward model operator g(m). The Levenberg–Marquardt‐lambda λ stabilizes the scheme; it is adapted according to the convergence of the iterations.

Because the covariance matrix $C_{M}$ is a full matrix, expressing both the prior uncertainty and correlation of the parameters, the first term in Equation 8 punishes both a deviation of the parameters from their priors and a variability that differs from that described by $C_{M}$, which is computed from the prior ensemble, resulting from geostatistical realizations. Hence, the first term of Equation 8 is a geostatistical regularization term. The other covariance matrix, $C_{D}$, expresses the trust in the measurements and accommodates correlated data errors that might stem from imperfect model conceptualization. In most applications, however, data errors are assumed to be uncorrelated so that $C_{D}$ becomes the diagonal matrix of measurement variances.

The inversion of covariance matrices in Equation 9 might become unfeasible for large models or data vectors. They are therefore typically replaced by low‐rank approximations.

In general $g\left(m_{j}\right)$ is non‐linear, so that Equation 9 needs to be applied several times, typically with decreasing values of λ until convergence. $C_{M}$ can easily be constructed from the ensemble of prior parameters. Unlike to traditional Gauss‐Newton schemes, G is also estimated from the ensemble as an average sensitivity $G^{e}$ between model predictions and parameters. This is achieved by approximate solutions of:

(10) $$\Delta d=G^{e}\Delta m$$

for example, by truncated singular value decomposition. Here, Δd and Δm are deviation matrices with $N_{e}$ columns containing realizations of predicted data and parameter vectors, respectively, minus their ensemble means. As a consequence, the number of model runs required for each update equals the number of realizations within the ensemble and is independent of the number of unknowns. However, a too small ensemble size for too many parameters may lead to a degenerate approximation of the Jacobian G.

We see the following advantages of the IES in the context of hydraulic tomography:

- Like all Bayesian methods, the IES inherently balances between meeting the observations and prior beliefs about the parameters.
- The posterior ensemble yields the conditional parameter uncertainty without relying on linearized uncertainty propagation.
- Due to successive linearization, the method remains robust in the face of (mildly) nonlinear predictive models.
- The IES stays applicable when parts of the model domain are ill‐posed and not sufficiently constrained by data due to its implicit geostatistical regularization. Parameters for which the data are hardly informative simply remain close to their initial ensemble.
- The IES stays applicable for large numbers of parameters and data as the computational requirements mostly depend on the number of realizations $N_{e}$.

In this study, the IES modifies the prior ensemble of log_10_(*K*) values to be consistent with observations within the assumed measurement error while obeying prior knowledge. The resulting conditional ensemble is referred to as the posterior ensemble.

For our computations we use *pestpp‐ies* (White, 2018) which is an exact implementation of the IES formulation by equation 18 of Chen and Oliver (2013) and part of the *PEST++* software suite (White et al. 2020).

## Synthetic Experiment

### Reference Field

The reference scenario is a bounded aquifer with a width of 100 m and a thickness of *m* = 10 m. Assuming negligible effect of vertical heterogeneity on the horizontal flow, we simulate a 2‐D domain. The log‐conductivity field is a second‐order stationary random space function with isotropic exponential covariance model, $\rho_{\mathrm{ref}}\left(d\right)=\exp\left(-d\right)$ (e.g., Webster and Oliver, 2007) with a characteristic length of 5 m. Note that we deliberately applied a different correlation function to generate the synthetic truth than the one used in inversion to mimic the typical lack of geostatistical knowledge in real world applications. The variance of log_10_(*K*) is 0.5 and the spatial geometric mean of hydraulic conductivity is 10^−4.5^ = 3.16 × 10^−5^ m/s. The field is generated by the randomization method (Kramer et al. 2007). The area of interest is 160 m long. We extended the computational domain by homogeneous buffer zones of 15 km length to avoid an influence of the inlet and outlet boundaries on the pumping tests. For the ambient hydraulic‐head field, we applied an overall hydraulic gradient of 3.5‰, by setting a constant‐head boundary condition 200 m upstream and downstream of the central control plane, resulting in a total discharge of 9.87 m^3^/d. We considered a uniform specific storage *S*_s_ of 1.0 × 10^−4^ m^−1^. Figure 1a shows the log‐conductivity field and the resulting hydraulic‐head field under steady‐state ambient‐flow conditions. Table 1 summarizes the quantities characterizing the reference field.

**Table 1:** Summary of Quantities Characterizing the Reference Field

Specific storage *S*_*s*_ (uniform)		1 × 10^−4^ m^−1^
Aquifer thickness (uniform)		10 m
Hydraulic conductivity *K* (structural parameters)	Spatial geometric mean of *K*	10^−4.5^ m/s = 3.16 × 10^−5^ m/s
	Variance of log_10_(*K*)	0.5
	Characteristic length of log_10_(*K*)	5 m
Flow field	Large scale hydraulic gradient	3.5 ‰
	Total discharge	9.87 m^3^/d

Note: The hydraulic‐conductivity field is a realization of a second‐order spatial random field with an isotropic exponential covariance function.

Even though the characteristic length of 5 m is significantly smaller than the domain, patches of smaller and larger conductivity values (with lengths of 20‐40 m) are present, which is typical for fields following an exponential covariance function. The hydraulic gradient is strongly affected by the presence or absence of conductivity variations that almost cover the entire width of the domain. We have chosen the reference case such that both heterogeneity and boundary conditions influence the ambient field and the pumping tests, which is typical for real world investigations.

### Experimental Design

The investigation targets are the total discharge through this aquifer and the variability of specific discharge across a transverse control plane in the center of the domain (dashed line in Figure 1a). We simulated 13 virtual pumping tests, using a different well as the pumping well in each test and observing the drawdown in all other wells, resulting in 13 × 12 = 156 drawdown curves. As shown in Figure 1b, nine wells are placed regularly within the control plane to obtain a good resolution of hydraulic conductivity therein. The remaining four wells are placed in a longitudinal transect. They are needed to obtain the ambient hydraulic gradient. We intentionally chose well spacings that are larger than the integral scale of the log‐conductivity field (5 m) because the length scales of heterogeneity are often poorly constrained in real world applications.

Ambient hydraulic‐head and transient drawdown data are simulated using *HydroGeoSphere* (Brunner and Simmons 2012). The simulated pumping rate is *Q*_Pump_ = 0.1 L/s over 3 h, followed by observation of the recovery, leading to a total simulated time of 320,000 s ≈ 3d 17 h. Small pumping rates were intentionally chosen to mimic a realistic field experiment using small‐diameter wells that can be installed with reasonable logistical effort (e.g., by utilizing direct‐push equipment, Leven et al. 2011).

We discretized the domain by an unstructured triangular mesh with a maximum element area of 1 m^2^. We added random measurement error, drawn from uncorrelated normal distributions with zero mean and a standard deviation of 0.01 m to the simulated drawdown values in the observation wells to obtain virtual measurements. In the inversion, we used 19 data points of each time series of drawdown (10 for the pumping and nine for the recovery phase), plus the ambient hydraulic‐head values at all wells, resulting in a total of 2977 data points.

### Definition of the Inverse Problem

In the inversion, we used a computational mesh of 9722 elements to parameterize the log‐conductivity field. To expedite the model runs, we coarsened the computational mesh compared to the reference model in the inversion calculations to a maximum element area of 2 m^2^ in the zone of interest. Specific storage was parameterized as a uniform value. Little is known about the spatial variability of *S*_*s*_ in real world aquifers, and we could have estimated it as a random space function. However, previous studies have found aliasing effects, where unresolved conductivity anomalies were wrongly attributed to anomalies in the specific‐storage field (Li et al. 2007). In light of little expert knowledge and the danger of aliasing, we assessed that keeping the storage coefficient constant was the conservative choice. The inversion parameters hence included the 9722 log‐conductivity values, a uniform storage coefficient, as well as the hydraulic‐head values of the ambient field at the in‐ and outflow boundaries for simulations of the ambient hydraulic‐head field. The latter hydraulic‐head values were applied as boundary conditions 200 m up‐ and downstream of the center of the investigation area.

### Initialization

To construct the prior ensemble, we generated unconditional realizations of log_10_(*K*) fields satisfying Equations (5), (6), (7) using the randomization method (Kramer et al. 2007). Because geostatistical structural parameters (*μ*_logK_, l, and *σ* ^2^_logK_) are typically unknown or at best uncertain in real world applications, we randomly sampled these parameters from assumed distributions. To obtain these distributions, we mimicked preliminary analyses of field data as performed by practitioners. To constrain the distribution of the mean conductivity, we simulated drawdown curves of single‐well pumping tests in the reference field and analyzed them following Papadopulos and Cooper Jr. (1967) to also account for wellbore storage effects. The diameter of wells is set to 0.05 m resulting in wellbore storage which is explicitly modeled as one‐dimensional pipe flow by *HydroGeoSphere*. The resulting distribution of log_10_‐conductivities has a mean of −4.4 and a variance of 0.38. We sampled the log‐normal distribution with these coefficients to obtain the mean hydraulic conductivity in the members of the prior ensemble. We chose not to use the analysis of single‐well drawdown curves for spatial conditioning of prior fields as it is difficult to attribute the correct sensitivity area of each curve a priori. Analogously, we sampled specific‐storage values from a log‐normal distribution (mean and variance of log_10_[*S*_s_]: −4.45 and 1.0, respectively). The variance of log_10_(*K*) was sampled from a uniform distribution between 0.2 and 2, and the characteristic length from a uniform distribution between 1.5 and 15 m. In each realization of the log‐conductivity field, we used a different set of geostatistical parameters drawn from the prescribed distributions. For simulations of the ambient‐head field, constant‐head values at the in‐ and outflow boundaries were sampled from a Gaussian distribution with variance 0.036 m^2^ and mean values 21.35 and 21.02 m of the upstream and downstream boundaries, respectively. These values stem from an extrapolation and uncertainty propagation of heads in the upper‐ and lowermost observation wells.

The prior ensemble consists of 600 realizations. We performed the ambient‐flow and pumping‐test simulations and picked the simulated hydraulic‐head values at observation times. This prior ensemble covers the full range of plausible states in parameter and data space to ensure that the sensitivity of the data on the parameters is covered by the ensemble, while expressing the prior uncertainty of the parameters. We express the data error of hydraulic‐head and drawdown, using a standard deviation of 0.01 m implicitly assuming the data error to be known and equal to the measurement error. In a real world experiment the appropriate data error magnitude is generally unknown and must be estimated with care to avoid compensation effects in the resulting model (overfitting). One possibility in the context of hydraulic tomography would be to compare reciprocal drawdown curves in the data which should in theory be identical. The observed differences should therefore represent a lower limit of how well an optimal model might be able to reproduce the obtained data. In our experiment we furthermore assume errors to be uncorrelated. Estimating error correlations alongside model parameters is suggested by several authors (e.g., Zhang et al. 2024). However, this requires sufficiently many correlated observations (Amezcua et al. 2023) which cannot be guaranteed because only a limited number of observations were used to describe each drawdown curve. Also, Amezcua and Van Leeuwen (2018) found that an overestimation of the correlation time scale worsens the results more than an underestimation. With practicality in mind, we assess the assumption of uncorrelated measurement error to be a reasonable and conservative simplification as also described by Doherty (2025). The measurement error is implemented in the inversion by perturbing the head/drawdown observations with random values drawn from a normal distribution with zero mean and standard deviation equal to the assumed measurement error. We iteratively updated the ensemble by employing *pestpp‐ies*. Misfit values did not improve notably after more than five iterations. With the chosen ensemble size of 600 we obtained a reasonable convergence and continuous posterior distributions, while keeping the computational effort within practical capabilities of a standard desktop computer (~250 core hours).

## Results

### Data Misfit

**Figure 2:**
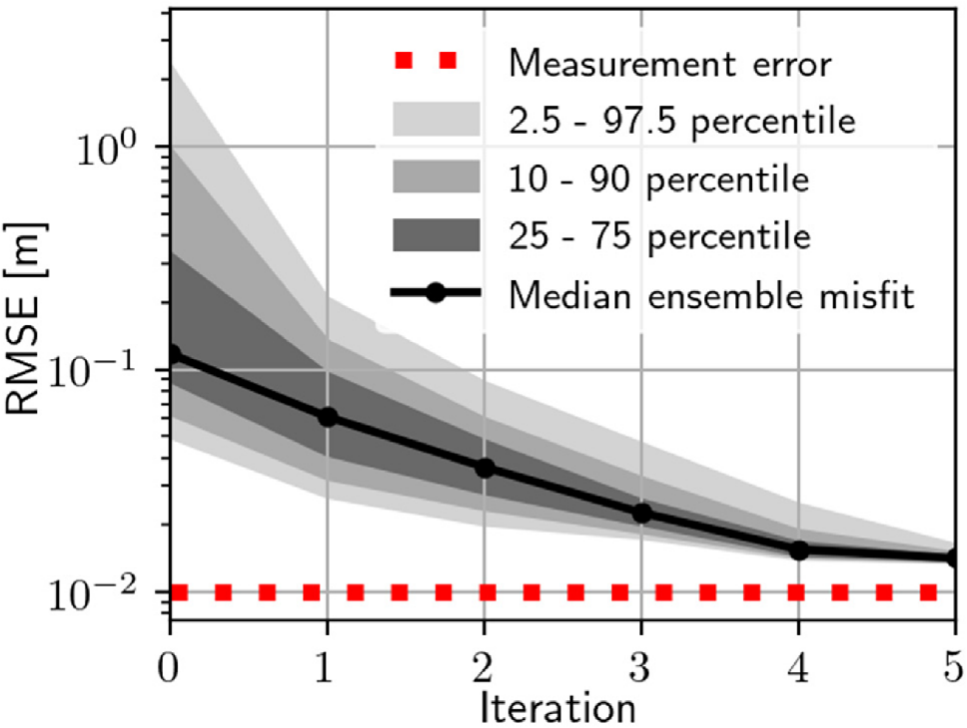
Root mean square error (RMSE) of simulated hydraulic‐head observations of all realizations over as function of the IES iterations compared to the assumed measurement uncertainty (red line).

**Figure 3:**
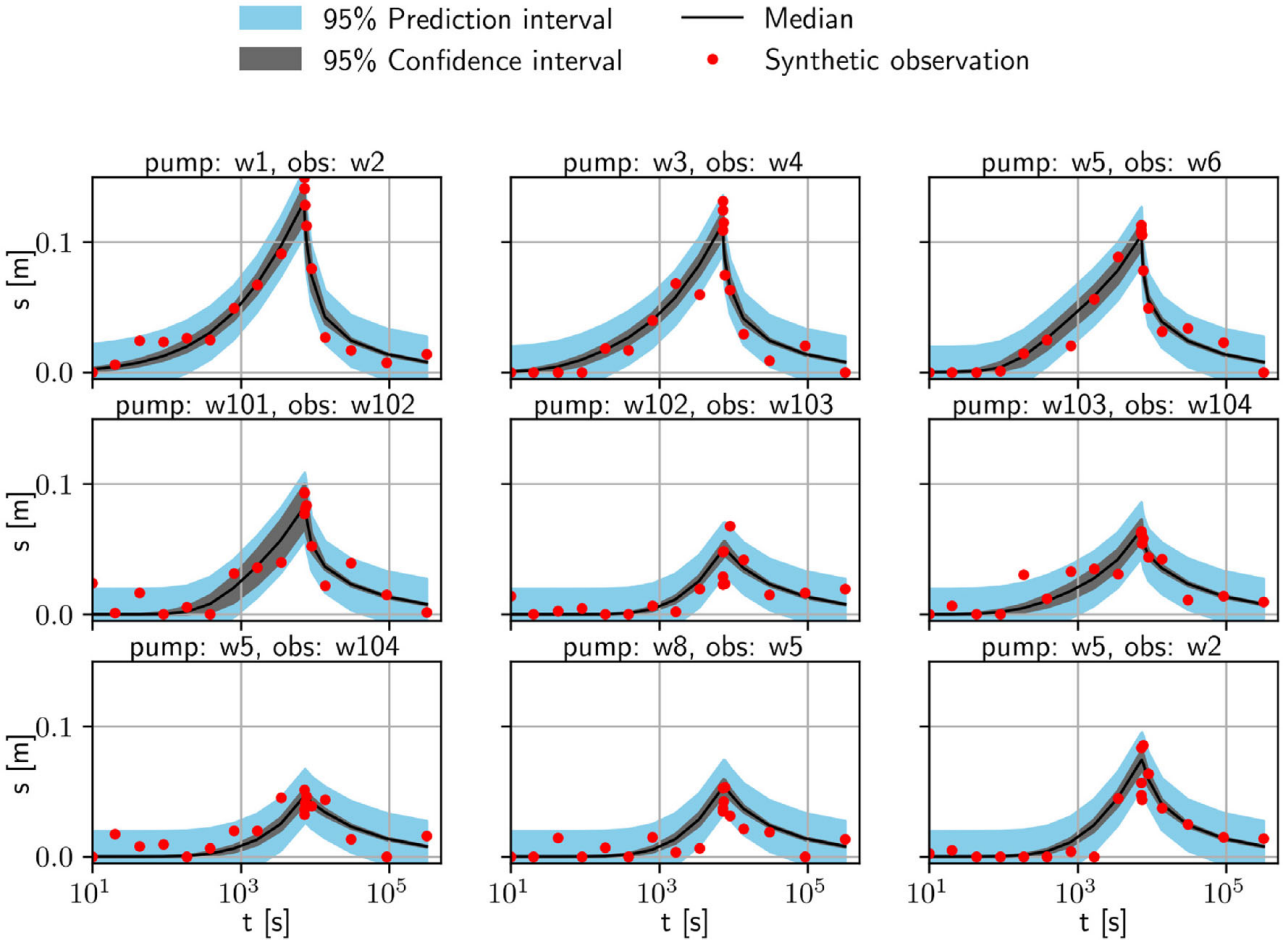
Drawdown over time after start of pumping of the posterior ensemble (gray band and black line) compared to synthetic reference data (red dots). Blue bands indicate the prediction interval. An exemplary selection of pumping (pump) and observation (obs) locations is shown here. The w‐numbers indicate the well positions in Figure 1b.

Figure 2 shows how the root mean square error (RMSE) of hydraulic heads evolves over the iterations of the IES inversion. The ensemble‐mean RMSE asymptotically approaches a value of 0.014 m which is reasonably close to the assumed measurement error of 0.01 m. This highlights the robustness of the IES scheme in the face of given non‐linearity. Figure 3 shows the comparison between disturbed synthetic observation data (red dots) and model outputs (black lines) of the posterior distribution for an exemplary selection of drawdown curves. The gray bands are 95% confidence intervals for the simulated drawdown curves. Blue bands show the 95% prediction interval, assuming a Gaussian distribution with variance equal to the sum of the measurement‐error variance (10^−4^ m^2^) and the confidence‐interval variance at each observation. The confidence and prediction intervals represent the posterior uncertainty in simulated true drawdown values and the expected probability of an (error‐prone) measurement to be observed, respectively. The overall shapes of the drawdown signals are reproduced well, and the vast majority of drawdown observations are located within the 95% prediction interval.

In general, the pumping phases show larger confidence intervals of drawdown than the recovery phases. The drawdown curves of individual conditional realizations typically over‐ or underestimate the drawdown at all times, implying strong positive correlation of drawdown for pumping only at different times. As the drawdown in the recovery period is the signal due to starting the pumping minus the same, time‐shifted signal due to stopping the pumping, positive correlations lead to an overall reduction of uncertainty. The underlying correlations are not perfect because the sensitivity volume of the observed head signal with respect to log‐conductivity increases with time. At late times all realizations must predict minimal drawdown, implying small uncertainty. But even the relative uncertainties of simulated drawdown decrease at late time because these values are more sensitive to large‐scale features, which are easier to infer.

### Posterior Conductivity and Hydraulic‐Head Fields

**Figure 4:**
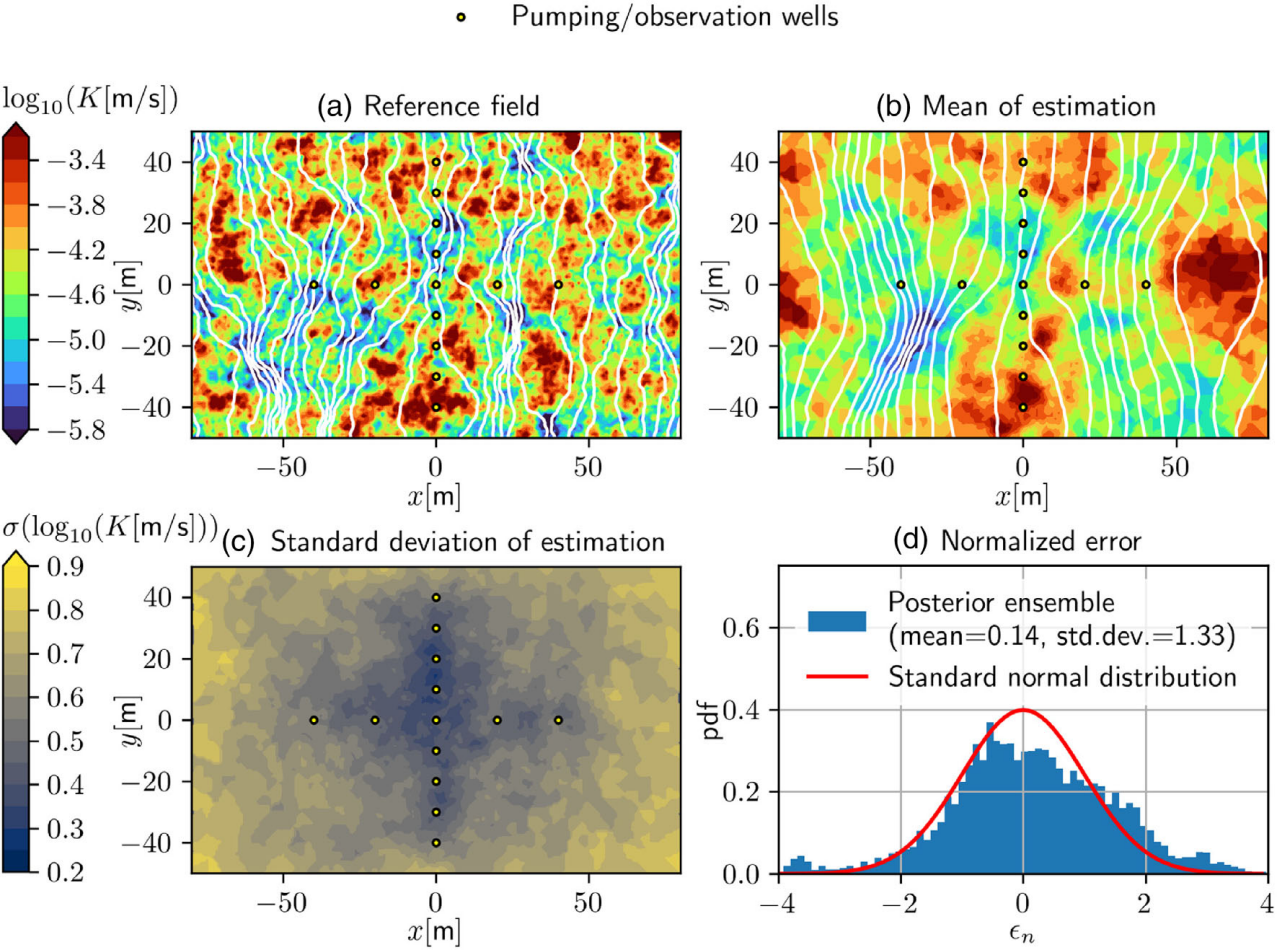
Log‐conductivity and hydraulic‐head fields (white contour lines) of the reference case and the inversion result. (a) reference case (same as Figure 1a); (b) posterior mean log‐conductivity field and corresponding head field; (c) standard deviation of estimation of posterior log‐conductivity; (d) empirical probability density function (pdf) of normalized error of log‐conductivity (Equation 11).

Figure 4b shows the ensemble means of the posterior log‐conductivity field and the ambient hydraulic‐head field in comparison to the corresponding reference fields (Figure 4a). The approach identifies large‐scale conductivity features in the vicinity of the wells, whereas fine‐scale features cannot be inferred from the data. With the well spacings (10‐20 m) being significantly larger than the underlying integral scales of log‐conductivity, this can be expected. The resulting spatial variations in hydraulic gradient (closeness of contour lines) are also visible. Figure 4c shows the standard deviation of estimation of log_10_(*K*) as a function of space. The posterior variability is the lowest close to the wells. This is expected because the sensitivity of hydraulic‐head observations to log‐conductivity is the highest close to the pumping and observation locations as demonstrated by Vasco et al. (2000). The posterior ensemble mean suggests two high‐*K* anomalies at the upstream and downstream ends of the domain, but this indication is untrustworthy since it falls outside the well coverage and its magnitude does not clearly exceed the local standard deviation of estimation. Also, these anomalies are not expected to significantly influence the relation between observed ambient heads and discharge since they lay outside of the well arrangement.

To further assess the quality of the posterior uncertainty we evaluated the normalized error ε_n_ of log‐conductivity in all finite‐element cells:

(11) $$\varepsilon_{n,j}=\frac{\mu_{e}\left(\log_{10}{\left(K\right)}_{j}\right)-\log_{10}{\left(K\right)}_{\mathrm{ref},j}}{\sigma_{e}\left(\log_{10}{\left(K\right)}_{j}\right)}$$

where the index *j* runs over the cells of the computational grid, whereas *μ*_*e*_ and *σ*_*e*_ are the ensemble mean and standard deviation of the posterior distribution, respectively. The subscript “ref” refers to the reference field. The empirical distribution of *ε*_*n*_, computed at all cells, describes whether the true conductivity value agrees with the posterior distribution. Ideally, if *ε*_*n*_ is uncorrelated and the assumptions of multi‐Gaussianity of the prior, likelihood, and posterior hold, *ε*_*n*_ follows the standard normal distribution, which expresses that the posterior mean is not biased (zero mean) and the estimation variance is well estimated (unit variance). Figure 4d shows the corresponding empirical distribution of *ε*_*n*_ which does not fully follow the standard normal distribution. The standard deviation is 1.33, the peak is too flat, and there are small secondary maxima at high and low normalized errors. This indicates a tendency for overconfidence which means that the absolute difference between the true and estimated values is often larger than the estimated uncertainty ranges. Nevertheless, given that the prior ensemble is not multi‐Gaussian, that we use the wrong correlation function with uncertain characteristic length, we assess the quality of the posterior ensemble to be within acceptable limits. Earlier iterations showed lower levels of overconfidence yet significantly higher biases (not shown).

### Estimation of Specific Discharge

The hydraulic conductivity field was fitted jointly with constant‐head values at the in‐ and outflow boundaries based on the drawdown and ambient‐head data. However, the drawdown data, which are insensitive to the large‐scale ambient flow field, dominated the data vector (2964 out of 2977 data points). Consequently, the boundary conditions were only updated marginally, which made an additional step necessary to estimate the ambient flow field. Upon convergence of the conductivity field, we fitted constant‐head values at the in‐ and outflow boundaries under ambient‐flow conditions to meet the ambient hydraulic‐head values. This is a linear inverse problem requiring no iteration. From that we computed the ambient specific discharge field **q**(**x**) and the total discharge $Q=\int_{-w/2}^{w/2}mq_{x}\left(x,y\right)\mathrm{dy}$ passing through the domain with width *w* and thickness *m*. As under ambient conditions we consider steady‐state flow without internal sources or sinks, the total discharge *Q* is the same at all values of the longitudinal coordinate *x*. When considering specific discharge, we analyze only the *x*‐component.

**Figure 5:**
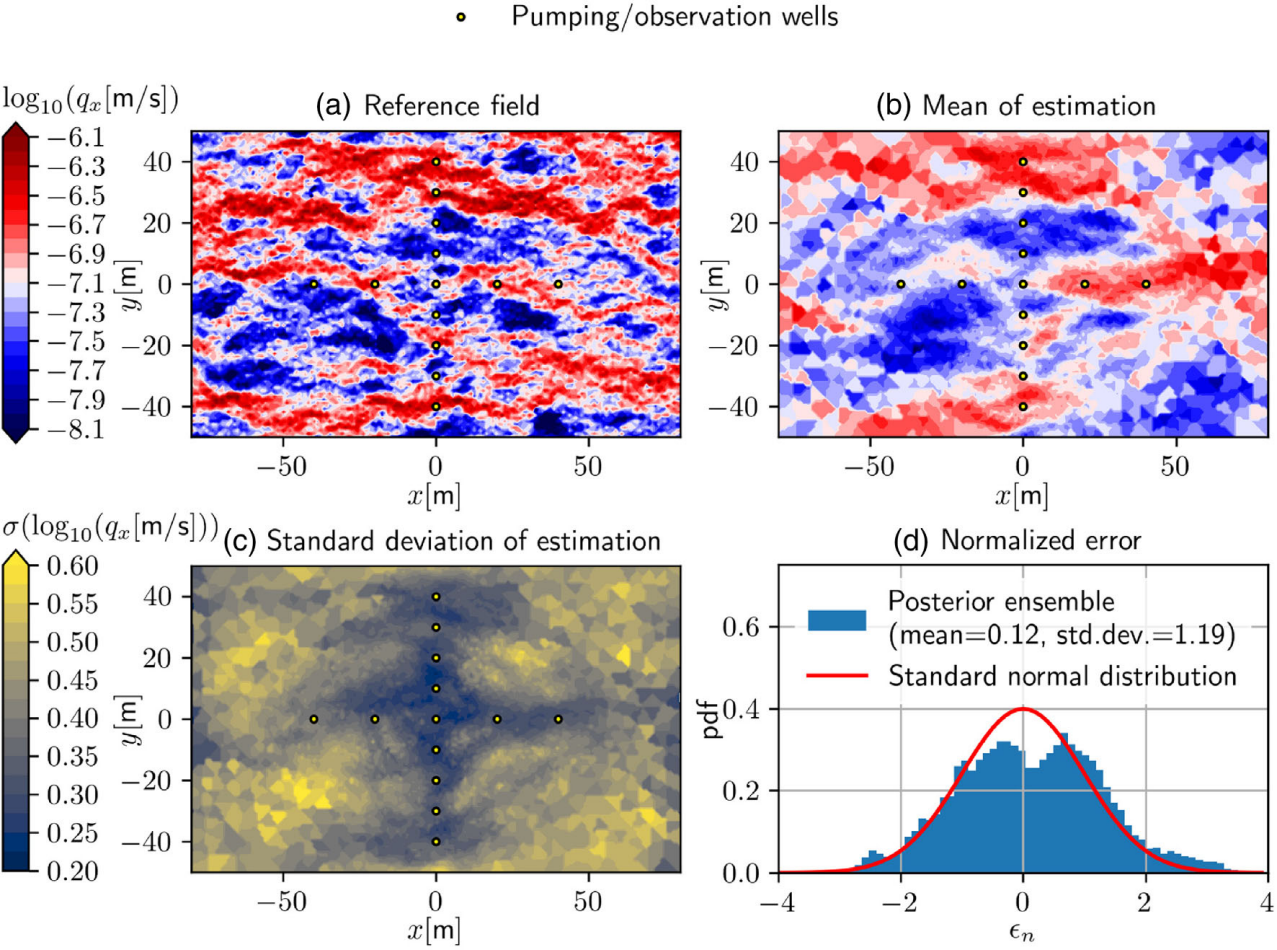
Specific discharge in x‐direction of the reference case and the inverted field. a) log_10_(q_x_) of the reference case; b) posterior mean of log_10_(q_x_); c) standard deviation of estimation of log_10_(q_x_); d): empirical probability density function (pdf) of the posterior normalized error ε_n_(log_10_(q_x_)) (analogous to Equation 11).

Figure 5 shows *q*_x_(**x**) of the reference field, the posterior mean, the standard deviation of estimation, and the *pdf* of the posterior normalized error of *q*_x_(**x**). The reference case (Figure 5a) shows streaks of elevated discharge oriented in the longitudinal direction. The location of those streaks crossing the well transect is well reproduced by the posterior mean (Figure 5b). The standard deviation of estimation for log‐velocity (Figure 5c) exhibits a similar pattern as that of log‐conductivity (Figure 4c), showing reduced uncertainty close to the wells. The posterior normalized error ε_n_(log_10_[*q*_x_]) indicates a weaker overconfidence (standard deviation of 1.19) and a better agreement with the standard normal distribution.

**Figure 6:**
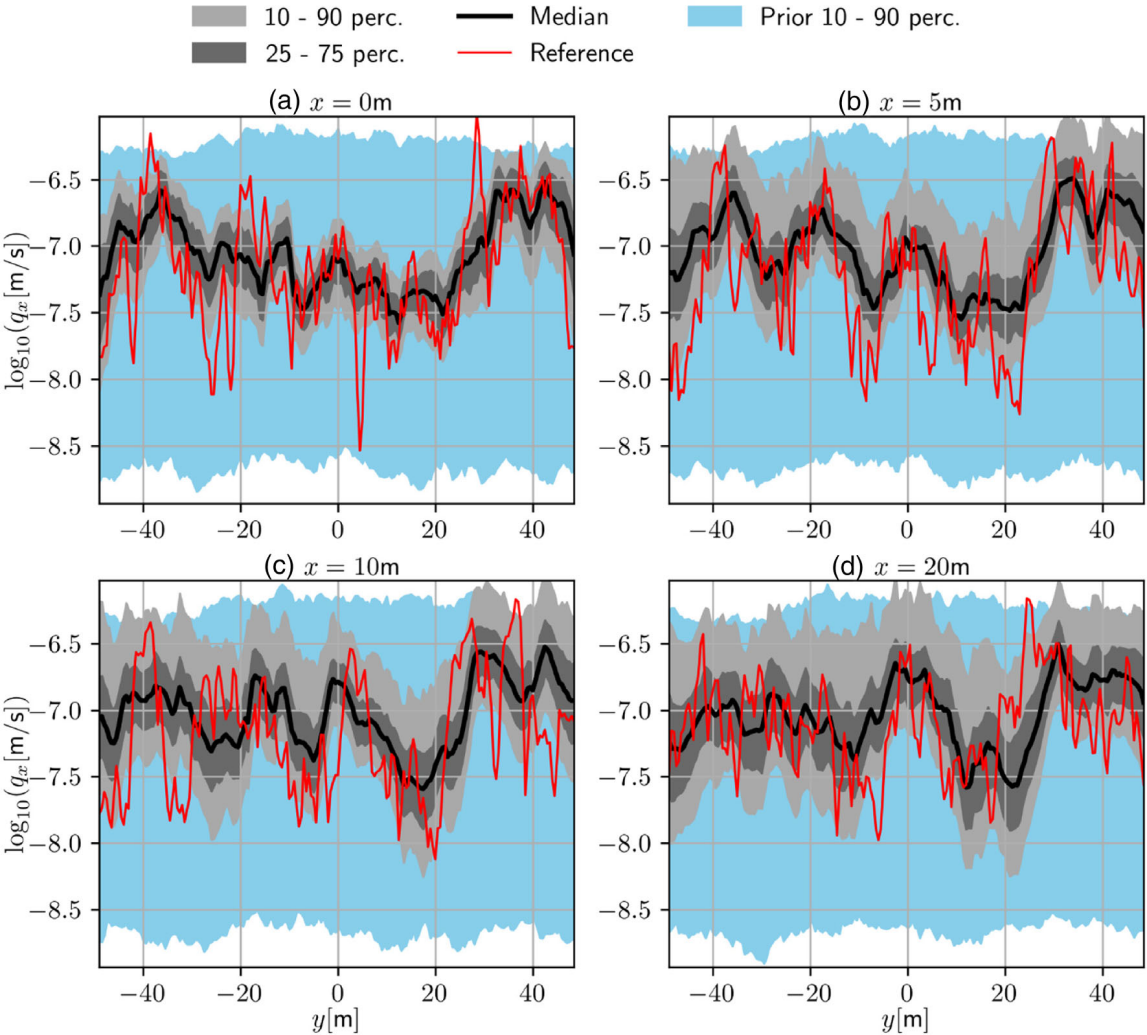
Specific discharge in x‐direction at the control plane at *x* = 0, *x* = 5 m, *x* = 10 m, and *x* = 20 m. Red: reference case; gray stripes: percentiles of the posterior ensemble; blue: 10‐90 percentile of the prior ensemble.

Figure 6 shows the profile of specific discharge *q*_x_ passing through the control planes at *x* = 0, x = 5 m, x = 10 m and x = 20 m. The reference case (red line) shows strong fluctuations at small spatial scales, which are mostly within the variability of the posterior ensemble. Larger‐scale trends of specific discharge are well recovered by the posterior median, whereas the small‐scale fluctuations are not uniquely inferred. That is, the individual conditional realizations (not shown) exhibit small‐scale fluctuations of *q*_x_ within the control plane, but they disagree on the location of local minima and maxima. In order to obtain a better identification of specific‐discharge fluctuations a denser well spacing would be needed (Vasco et al. 1997), which we consider unrealistic under field conditions. The ensemble variability increases with increasing longitudinal distance from the central control plane (*x* = 0), but the median still resembles the true anomalies even at *x* = 20 m. These results indicate that specific discharge is resolved not only at the defined control plane (*x* = 0 m) but also in its neighborhood. This is consistent with specific discharge being correlated over longer distances in the direction of mean flow than hydraulic conductivity (e.g., Rubin 1990). While the misfit between the estimated and true specific‐discharge profiles increases with distance to the control plane, the ensemble variability also increases.

Overall, we assess the posterior ensemble of specific discharge to be more robust concerning anomaly detection and uncertainty estimation than the posterior ensemble of log‐conductivity (comparing Figures 5d and 4d, respectively). This is somewhat surprising given that ambient specific discharge has not been part of the calibration process and does not influence the drawdown data used in inversion at all. However, it is well known from linear stochastic theory, that the variance of longitudinal log‐velocity is substantially smaller than that of log‐conductivity because part of the variability in conductivity results in variability of the hydraulic gradient (e.g., Rubin 1990). This smoothing mechanism may also explain why the local tendency to be overconfident in estimating conductivity does not carry over to the overconfidence in estimating specific discharge to the full extent.

### Estimation of Total Discharge

#### Tomography‐Based and Conventional Approaches

From the posterior ensemble of the ambient specific‐discharge field (Figures 5 and 6), we obtained the corresponding ensemble of total discharge by integration over the cross‐section perpendicular to the *x*‐direction. To assess the effect of well spacing, we generated a second specific‐discharge ensemble for a reduced data set where we used only w1, w3, w5, w7, w9, w101, and w104 for pumping and observation. We compared these two ensembles to a conventional approach of estimating total discharge based on an analytical evaluation of measured drawdown curves.

To estimate the total discharge using a more traditional approach, we considered the late‐time behavior of the Theis (1935) solution:

(12) $$T=\frac{Q_{\text{pump}}}{4\pi d},$$

where, *d* is the derivative of drawdown with respect to the natural logarithm of time, ln(*t*):

(13) $$d=\frac{\partial s}{\partial \ln\left(t\right)}=t\frac{\partial s}{\partial t}.$$

When the underlying assumptions (homogeneous transmissivity and no influence of boundaries) are effectively fulfilled, the log‐time derivative of drawdown should approach a constant value at very late times. This phase is also referred to as infinite‐acting radial flow. In heterogeneous aquifers such behavior will occur once the sensitivity volume of drawdown is large enough to be representative (Sanchez‐Vila et al. 1999). No‐flow boundaries lead to an increase of the log‐time derivative at late times once the cone of depression has reached them (e.g., Walker and Roberts 2003). It is, therefore, best practice to determine different regimes of drawdown curves with respect to their log‐time derivatives prior to a quantitative analysis (e.g., Renard et al. 2009). An infinite‐acting radial flow regime is characterized by a plateau of the log‐time derivative. Effective transmissivity is subsequently obtained by applying Equation 9 using the value of that plateau.

**Figure 7:**
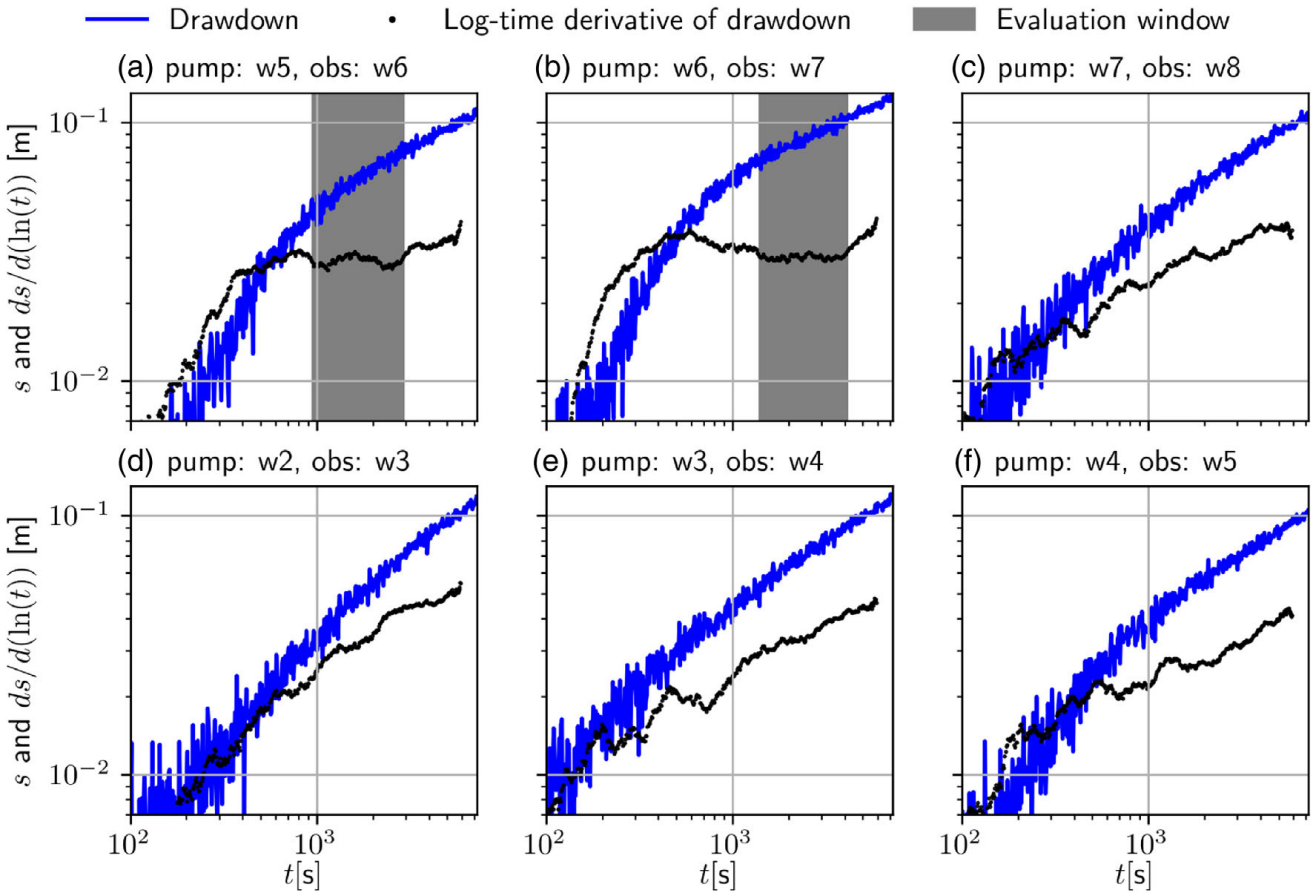
Drawdown curves (blue) and their log‐time derivatives (black dots) for six well pairs as a function of time. (a,b) Examples showing a regime with constant log‐time derivatives (highlighted by gray areas) that can be analyzed to obtain effective transmissivity values; (d‐f) examples not showing such behavior. The w‐numbers indicate the well positions in Figure 1b.

For the given case we considered the drawdown data of neighboring well pairs as those curves showed the best pronunciation of different flow regimes. In order to mimic measurement error, we added random noise to the data drawn from a normal distribution with zero mean and a standard deviation of 0.003 m. This value is intentionally chosen smaller than that assumed in the tomographic approach (0.01 m) as only temporal changes evaluated over a short time window enter the evaluation (Equation 12). In a real measurement such changes are expected to be more robust than absolute measurements of drawdown which are additionally affected by systematic bias and long‐time instrumental drifts. We computed the log‐time derivative employing the method of Spane and Wurstner (1993). In this method, the derivative at time *t*_*i*_ is calculated by performing a linear regression using all value pairs of *s* and log(*t*) recorded during the interval [log(*t*_*i*_)‐*L*, log(*t*_*i*_) + *L*]. We chose an *L*‐value of 0.4. Figure 7 shows an exemplary selection of log‐time derivate curves of drawdown d as function of time in double logarithmic plots. Only two drawdown curves (Figure 7a and 7b), using wells in the center of the domain (wells 5‐7 according to Figure 1b), show a regime with a short constant log‐time derivative d. These plateaus are followed by a regime, in which the curve of log(d) as function of log(t) has a slope of approximately 1/2, indicating an effective flow dimension of one (Chakrabarty 1994), caused by the two parallel no‐flow boundaries. The gray areas in Figure 7a and 7b indicate the time windows with an approximately constant log‐time derivative. The log‐time derivative values in these windows were used to estimate effective transmissivities using Equation 12. In the case of the other four drawdown curves (Figure 7c through 7f), the early‐time regime with increasing log‐time derivative directly transitions into the late‐time regime affected by the boundary conditions. These tests don't show an infinite‐acting radial‐flow regime at all and cannot be analyzed by Equation 12.

In a second step, we multiplied the obtained effective transmissivity with the channel width and a uniform hydraulic gradient obtained from the ambient‐head measurements in the two outmost wells w101 and w104 to estimate total discharge. As log‐time derivative values vary within the analyzed time windows, we obtained an ensemble of total‐discharge estimates by conventional analysis. This analysis represents an example of the best‐practice technique including the diagnostics for the identification of infinite‐acting radial‐flow regimes under optimistic conditions (noise with standard deviation of only 0.003 m). It is equivalent to applying the standard straight‐line method using semi‐logarithmic drawdown curves if the linear regression is restricted to the part of the plot that is really linear. Disregarding nonlinearity of the analysis of much noisier data would realistically worsen the results. Hence, the traditional data analysis shown here is meant as the best possible conventional benchmark to compare against our much more elaborate and costly tomographic approach.

#### Comparison of Approaches

**Figure 8:**
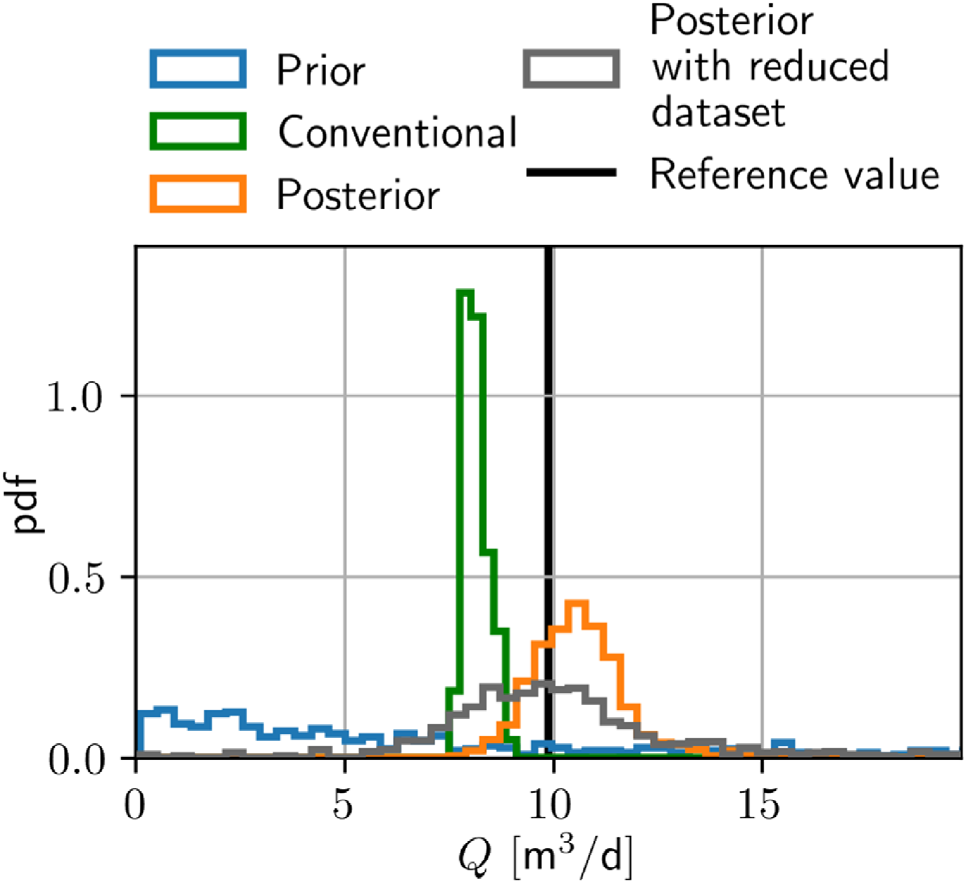
Prior and posterior ensembles of total discharge compared to the conventional estimation using the log‐time derivative (Figure 7a and 7b). The vertical line shows the reference value.

Figure 8 shows the histograms of the prior (blue) and posterior (orange) ensembles of total discharge as well as the discharge estimates obtained by the conventional approach (green). The reference value, *Q*_True_ = 9.87 m^3^/d, is shown as a vertical black line. The results based on a reduced dataset (utilizing only w1, w3, w5, w7, w9, w101, and w104) are shown in gray.

The tomographic and conventional approaches give values in the correct order of magnitude with (median values of 10.54 and 8.08 m^3^/d, respectively) but differ notably when compared by their mean absolute errors (MAE) which are 1.19 and 1.72 m^3^/d, respectively. The tomographic approach with a reduced data set gives by far the largest MAE of 4.62 m^3^/d. While the true value is only marginally included within the estimated uncertainty of the conventional approach, the tomographic approach shows a more plausible uncertainty of total discharge. We quantified this by the *Continuous Ranked Probability Score* (CRPS) of the distributions defined as Hersbach (2000):

(14) $$\text{CRPS}=\int_{\infty }^{\infty }{\left(F\left(Q\right)-H\left(Q-Q_{\text{true}}\right)\right)}^{2}\mathrm{dQ},$$

where *F*(*Q*) is the empirical cumulative distribution function of total discharge *Q* and *H*(·) is the Heaviside function. The CPRS provides a unified metric that includes both the accuracy of a probabilistic prediction and the narrowness of the distribution. Lower values indicate a better prediction. The posterior distribution obtained from the tomographic approach gives a CRPS of 0.40 m^3^/d whereas the conventional approach yields a value of 1.65 m^3^/d. When only a reduced data set is used for a tomographic analysis the CRPS remains similar at 0.55 m^3^/d despite the larger MAE. Hence, the tomographic method is more reliable in estimating total discharge and in particular the associated uncertainty than the conventional approach. Nonetheless, the conventional approach using the Theis solution proved to be a close approximation of total discharge which is consistent with the findings of other authors (Sanchez‐Vila et al. 1999; Larocque et al. 2009). Sections of the drawdown curves with constant log‐time derivative must therefore indeed be dominated by the effective transmissivity despite the existence of borders. We attribute the difference between the mean estimated discharge and the reference value (8.15 and 9.87 m^3^/d, respectively) to a potential non‐representativeness of the sensitivity volumes before boundary effects start to influence the data.

A drawback of the conventional pumping‐test analysis in the given example is that only a minority of drawdown curves (2 out of 156 obtained from 13 pumping tests) showed a regime with constant log‐time derivative, whereas in the remaining curves the boundary effects became dominant before a plateau in the log‐time derivative was reached. In real world applications where the subsurface structure is unknown a priori, it would therefore be unlikely to obtain robust data without installing a similar number of wells as in our virtual experiment. Additionally, the potential non‐stationarity of the underlying transmissivity field might introduce further ambiguous characteristics in the drawdown curves. Considering the added value of a more reliable uncertainty estimate and resolving part of the heterogeneity in log‐conductivity and specific discharge, we assess the tomographic approach to be superior to the conventional analysis method for the given set of drawdown curves, despite the larger modeling and inversion effort. This is also the case when only a coarse well arrangement with fewer drawdown curves can be utilized. While the uncertainty is significantly larger than for a finer well spacing, it correctly reflects the limitations of the respective data coverage.

## Discussion and Conclusions

The discharge‐targeted hydraulic‐tomography approach proposed in this study estimates the total discharge passing through a control plane with a reasonable uncertainty range, whereas it seems to be slightly overconfident in inferring the spatial distributions of hydraulic conductivity and specific discharge. However, the approach is not designed to infer these fields at larger distances to the control plane. For the latter, a truly two‐dimensional tomographic approach, at best with a spacing of wells on the order of the characteristic length of heterogeneity, would be adequate. Such a survey would involve a much higher experimental effort in a field application than our method and may be considered unrealistic in field applications outside of research projects. Hence, the issue of the observed overconfidence is not so much that our approach yields erroneous values of log‐conductivity and specific discharge far away from the control plane, but that the scheme yields a too small standard deviation of estimation.

We conjecture that this tendency to overconfidence is a local effect, arising from imperfect spatial resolution and imprecise localization of anomalies rather than generally wrong identification of those anomalies. This becomes apparent when comparing Figures 5b and 6: While the overall shapes of anomalies in the discharge field are well captured (Figure 5b), local maxima as well as the bounds of anomalies can be shifted in space (Figure 6), resulting in locally strong differences and hence larger normalized errors *ε*_*n*_ (Figures 4d and 5d). This may be analyzed in detail by applying a more elaborate way of calculating the normalized error that accounts for spatial correlation of the misfit, or by comparing values of the reference field with those of the inferred field within a spatial window, as done by the structural similarity index used in image comparison (SSIM, Zhou et al. 2004). Such analyses, however, were beyond the scope of our study.

In our application of the IES, we considered one log‐conductivity value per element of the computational grid plus one storativity value and two values of hydraulic head at boundaries, resulting in 9725 parameters to be estimated from 2977 hydraulic‐head and drawdown measurements. Neither the number of parameters nor that of the data points caused computational limitations of the IES. We also tested a version, in which we parameterized the log‐conductivity field as pilot‐point based conditional realizations (Certes and deMarsily, 1991; RamaRoa et al., 1995), using 89 and 173 pilot points, respectively (results not shown). With the smaller numbers of pilot points, we could not fit all drawdown curves with sufficient accuracy, obviously because we did not place pilot points into all sensitive regions. In both pilot‐point applications, the conditional uncertainty of the log‐conductivity field showed erroneous minima in elements containing a pilot point, thus reflecting the choice of pilot‐point locations rather than the sensitivity of the drawdown curves to the log‐conductivity field. This artifact of the pilot‐point method is well known in geostatistical inversion and did not occur with the approach presented here. Neither the number of iterations needed to achieve convergence of the log‐conductivity field, nor the computational effort differed significantly between the pilot‐point based inversions and the approach with one log‐conductivity value per element of the computational grid. This is an inherent property of the IES method, in which the computational effort depends mainly on the number of realizations, rather than that of the parameters or measurements. Thus, unlike other authors who recently have used the combination of pilot points and the IES in calibrating groundwater models (e.g., Cao et al. 2018; Hayley et al. 2019; Farnum et al. 2025), we don't see any need or benefit of using pilot points.

It was difficult to infer the specific‐discharge variation within the control plane with high precision because the chosen well spacing was larger than the characteristic length of heterogeneity. However, we wanted to mimic a realistic field survey. To cover the entire aquifer width of 100 m, we already chose nine wells at a spacing of 10 m, plus four wells oriented in the direction of the valley to infer the mean ambient hydraulic gradient. We consider this to be at the upper limit with respect to experimental effort when it comes to field applications. Finding the balance between desired resolution and affordability will always remain a challenge in hydrogeological field surveys. While it can be suspected that for a given well spacing the resolution of mapped anomalies will be on a similar length scale, it is less clear how low the resolution is allowed to be to produce a satisfactory estimate of total discharge. This question is part of ongoing work by the authors. Including drawdown observations from a limited number of wells to improve inferred conductivity fields is furthermore subject to current research (e.g., Manewell et al. 2024).

Our tomographic approach did an excellent job in estimating the total discharge. Practitioners might ask whether the effort of a tomographic survey is justified. This is why we compared our estimate to that obtained by using two wells for a traditional pumping test, analyzed by the late‐time solution of the Theis approach, and two wells to obtain the hydraulic gradient. Because of the two no‐flow boundaries, the vast majority of drawdown curves (154 out of 156) could not be analyzed in this way. The two remaining drawdown curves yielded total‐discharge estimates that differed more significantly from the reference value than the estimate by the tomographic survey and provided no good uncertainty estimate. While it is no surprise that only well pairs in the center of the channel‐like aquifer could be analyzed under the assumption of an infinite‐acting radial‐flow regime, there would be no guarantee that an arbitrarily chosen well pair would meet that requirement.

Our synthetic test case was somewhat idealized: The chosen log‐conductivity field was a second‐order stationary multi‐Gaussian field (even though the geostatistical parameters were not known in the inversion); we neglected vertical variation of log‐conductivity, there was no exchange with a surface‐water body, and no lateral inflow from adjacent hillslopes. Whether contributions to the water balance from a larger number of boundaries can be reliably investigated based on a tomographic conductivity field will be evaluated by future applications of the method in real world applications.

Overall, this study demonstrated that estimating groundwater discharge on a spatial scale (100 m) that is realistic for field applications can be reliably achieved with a targeted tomographic approach. We propose a design that can be realized with a feasible number of field installations that can be implemented, for example, by using direct‐push equipment. Due to the efficiency of the IES, the computational effort is affordable to consultancies, and the results include decent uncertainty estimates. Given that the tomographic approach targets total discharge, the estimates of spatial variability of hydraulic conductivity and specific discharge may be seen as extra information gained by the approach.

## Data Availability

The model files, pest++ control files, synthetic data, results, and postprocessing scripts are made available at https://doi.org/10.57754/FDAT.kacah‐cas88.
